# Supplementary material for: Transcriptional Regulator DasR Represses Daptomycin Production through Both Direct and Cascade Mechanisms in Streptomyces roseosporus
Source: Antibiotics (Basel). 2022 Aug 5;11(8):1065. doi: 10.3390/antibiotics11081065 (PMC9404778; doi:10.3390/antibiotics11081065)
Supplement: Supplementary file 1 [file antibiotics-11-01065-s001.zip › antibiotics-1818174-supplementary.pdf]

Supplementary Material

Transcriptional regulator DasR represses daptomycin production through both direct and cascade mechanisms in *Streptomyces roseosporus*

Qiong Chen[1#], Jianya Zhu[2#], Xingwang Li[1], Ying Wen[1*]

[1] State Key Laboratory of Agrobiotechnology and College of Biological Sciences, China Agricultural University, Beijing 100193, China

[2] Institute of Fisheries Research, Beijing Academy of Agriculture and Forestry Sciences, Beijing 100068, China

# These authors contributed equally to the study.

* Correspondence: wen@cau.edu.cn (Y.W.); Tel: +86-10-62732715

Figure S1.

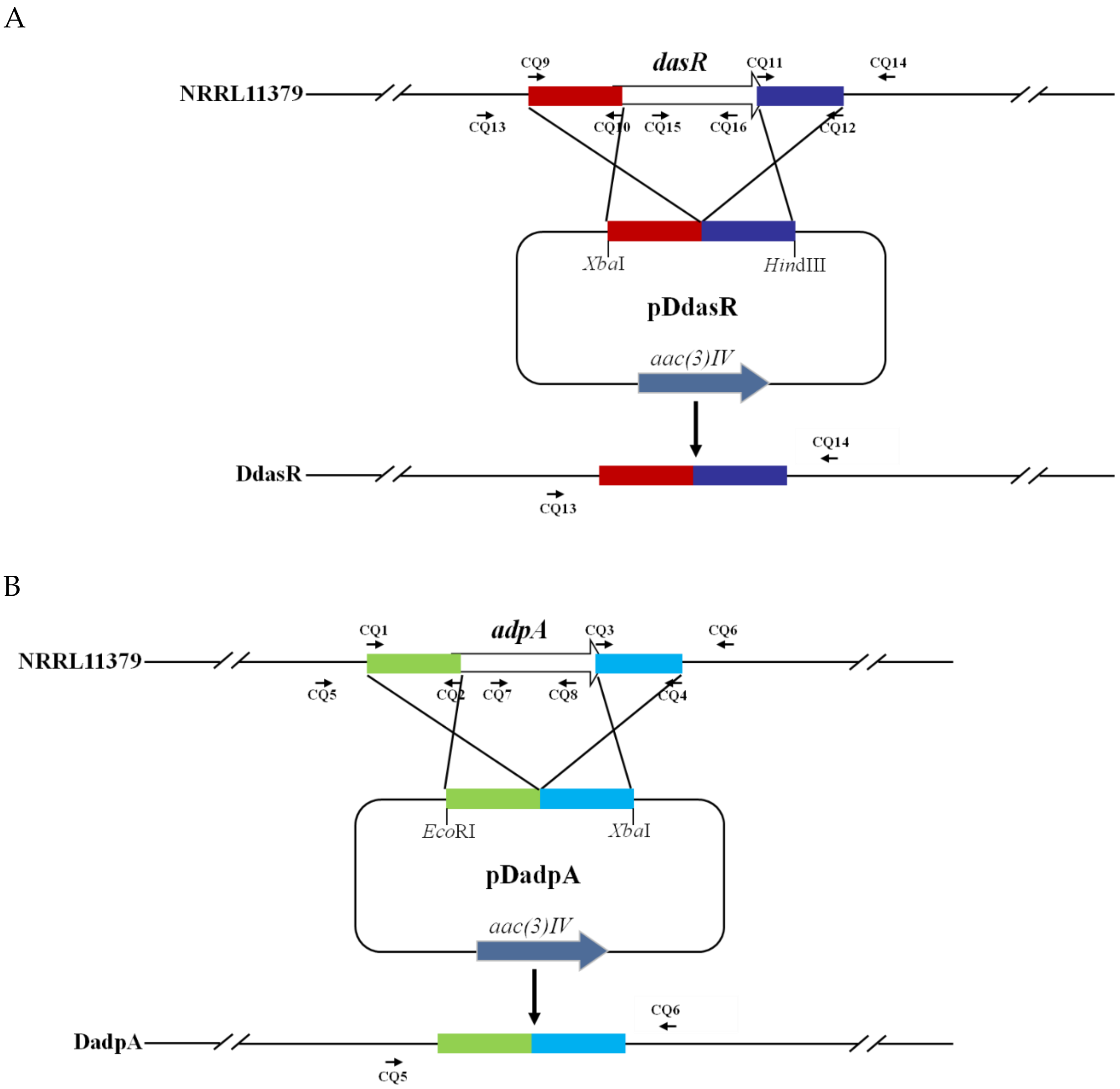

Figure S1. Strategy for deletion of *dasR* (**A**) and *adpA* (**B**) in WT (schematic). Large arrows: genes and their directions. Small arrows: positions of primers. Rectangles: homologous exchange regions used for gene deletion.

Figure S2.

A

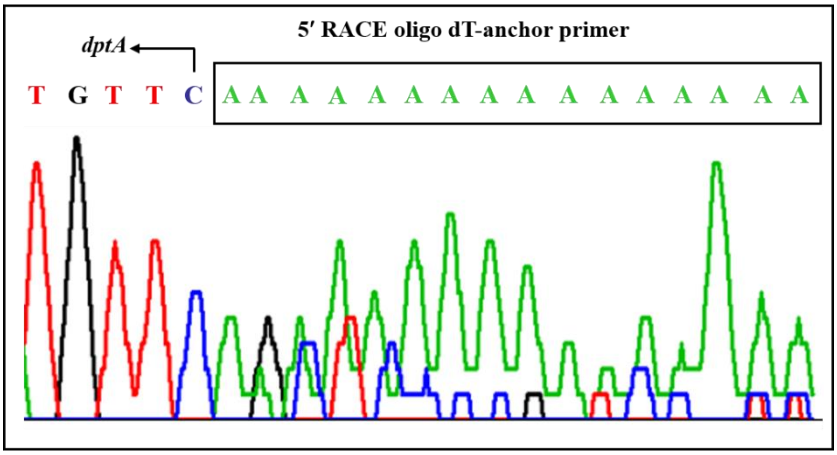

B

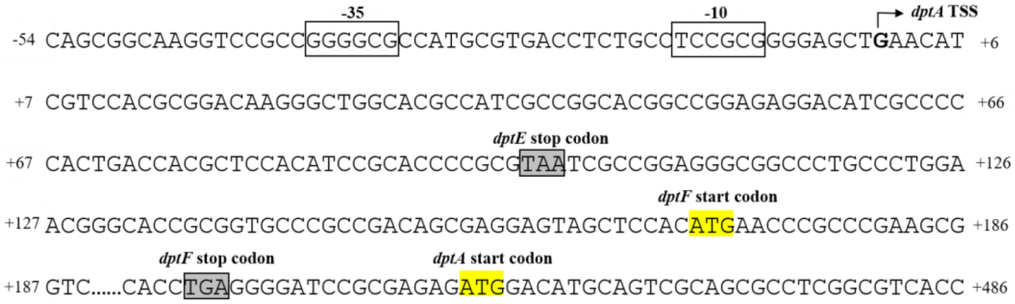

Figure S2. Determination of *dptA* TSS. (**A**) 5′ RACE analysis of *dptA* TSS. Box: complementary sequence of oligo dT-anchor primer. Bent arrow: complementary base of TSS. (**B**) Promoter structure of *dptA*. Boxes: putative −10 and −35 regions. Bent arrow: *dptA* TSS. Yellow highlight: TSCs. Shading: translational stop codons.

Figure S3.

A

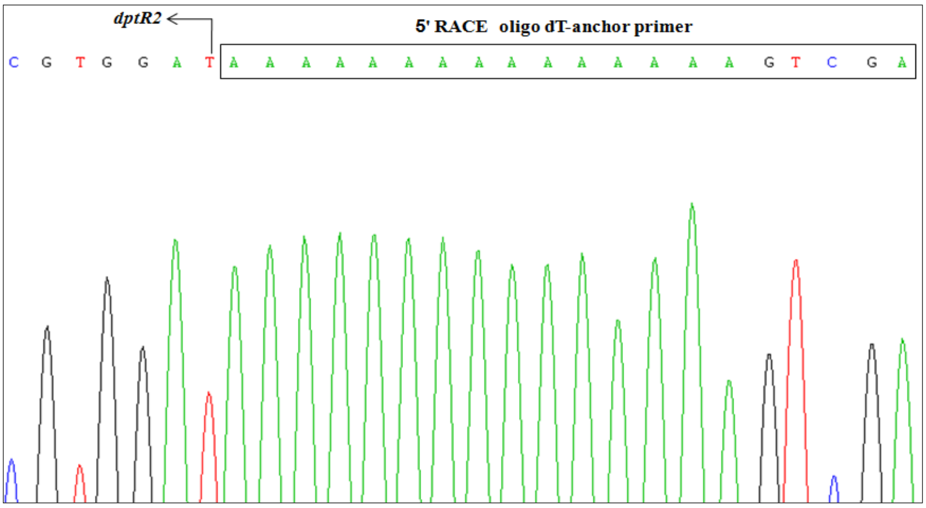

B

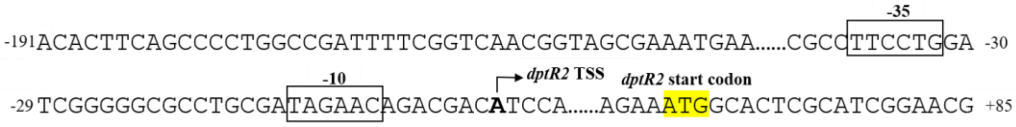

Figure S3. Determination of *dptR2* TSS. (**A**) 5′ RACE analysis of *dptR2* TSS. Box: complementary sequence of oligo dT-anchor primer. Bent arrow: complementary base of TSS. (**B**) Promoter structure of *dptR2*. Boxes: putative −10 and −35 regions. Bent arrow: *dptR2* TSS. Yellow highlight: *dptR2* TSC.

Table S1. Strains and plasmids used in this study.

Strains or plasmids	Description	Source or reference
**Strains**		
S. roseosporus		
NRRL11379	WT strain, daptomycin producer	Laboratory stock
DdasR	*dasR* deletion mutant	This study
CdasR	*dasR* complemented strain	This study
OdasR	*dasR* overexpression strain	This study
DadpA	*adpA* deletion mutant	This study
CadpA	*adpA* complemented strain	This study
OadpA	*adpA* overexpression strain	This study
WT/pKC1139	WT strain carrying empty vector pKC1139	This study
WT/pSET152	WT strain carrying empty vector pSET152	This study
E. coli		
JM109	General cloning host	Laboratory stock
ET12567	Methylation-deficient strain	[1]
BL21 (DE3)	Host for protein overexpression	Novagen
**Plasmids**		
pKC1139	Multiple-copy *E. coli-Streptomyces* shuttle vector	[2]
pSET152	Integrative *E. coli-Streptomyces* shuttle vector	[2]
pET-28a (+)	Vector for His_6-tagged protein overexpression in *E. coli*	Novagen
pJL117	pIJ2925 derivative carrying promoter *ermE*p*	[3]
pDdasR	*dasR* deletion vector based on pKC1139	This study
pCdasR	*dasR* complemented vector based on pSET152	This study
pOdasR	*dasR* overexpression vector based on pKC1139	This study
pET28-dasR	His_6-DasR overexpression vector based on pET-28a (+)	This study
pDadpA	*adpA* deletion vector based on pKC1139	This study
pCadpA	*adpA* complemented vector based on pSET152	This study
pOadpA	*adpA* overexpression vector based on pKC1139	This study
pET28-adpA	His_6-AdpA overexpression vector based on pET-28a (+)	This study

Table S2. Primers used in this study.

Primer	DNA sequence * (5'-3')	Purpose
CQ9	GCTCTAGATTTCCTTGCGGTCCTTCGG (*Xba*I)	Deletion of *dasR* gene
CQ10	CTTCAGACGCGCCACGAAGGTCTCGCTCCCCGTACT	
CQ11	AGTACGGGGAGCGAGACCTTCGTGGCGCGTCTGAAG	
CQ12	CCCAAGCTTCCCGTCGGTCGGTGGGAT (*Hin*dIII)	
CQ13	CGAGGGAGGTGTCCAGCTTC	Confirmation of *dasR* deletion in mutant DdasR
CQ14	CGTCGCGCGCCATCTGAC	
CQ15	ACCTTCGTCGCCAAGCCG	
CQ16	GGGACAGCATGAGCATCGG	
CQ21	CCCAAGCTTACGCAAGCGTCGGGAAGG (*Hin*dIII)	Overexpression of *dasR* in *S. roseosporus*
CQ22	GCTCTAGACGGTTCACGTGATCCTGC (*Xba*I)	
CQ75	CGGAATTCCCCACCGCGAAGACATTC (*Eco*RI)	Complementation of *dasR* in DdasR
CQ76	GCTCTAGAGTTCACGTGATCCTGCTCC (*Xba*I)	Overexpression of His$_6$-DasR in *E. coli*
CQ25	CGGAATTCAGTACGGGGAGCGAGACC (*Eco*RI)	Deletion of *adpA* gene
CQ26	CCCTCGAGCGGTTCACGTGATCCTGC (*Xho*I)	
CQ1	CGGAATTCTGACCCCTTCGCGGGATG (*Eco*RI)	
CQ2	GGAACGAACGCGTCAGGATGAACAGCAGCACGGCAA	
CQ3	TTGCCGTGCTGCTGTTCATCCTGACGCGTTCGTTCC	
CQ4	GCTCTAGAGTCACGGATGTCCGCCAG (*Xba*I)	
CQ5	ACACGCCATGAGGCCGC	Confirmation of *adpA* deletion in mutant DadpA
CQ6	CACAACCAGGTGCTCTGCC	
CQ7	GAACTCACCACGCCGTACG	
CQ8	GCACATCGCCGTCGTCCA	Overexpression of *adpA* in *S. roseosporus*
CQ17	CCAAGCTTCACCGAGGGGGGCTTAGC (*Hin*dIII)	
CQ18	GCTCTAGAGCGGGCGGATCACGATGT (*Xba*I)	
CQ19	CGGAATTCTGACCCCTTCGCGGGATG (*Eco*RI)	Complementation of *adpA* in DadpA
CQ20	GCTCTAGAGCGGGCGGATCACGATGT (*Xba*I)	Overexpression of His$_6$-AdpA in *E. coli*
CQ23	CGGAATTCATGAGCCAGGACTCCGCC (*Eco*RI)	Amplification of *adpA* ORF for RT-qPCR
CQ24	CCCTCGAGCGCAGTCGATCCACACCAT (*Xho*I)	
CQ27	GCTTAGCGTCATGAGCCAG	
CQ28	TGAACAGCAGCACGGCAA	
ZQL75	TCCCGGGAATCGCGAGAG	Amplification of *dptR3* ORF for RT-qPCR
ZQL76	CCCGGATTCCCCCGACTC	
ZQL79	GAACAGACCACCCTCCTCG	Amplification of *dptBC* ORF for RT-qPCR
ZQL80	CTGTGGCCGATGGGGTAG	
ZQL81	CGGCGTACATCATCCAGACC	Amplification of *dptA* ORF *for* RT-qPCR
ZQL82	GTCATGCTCAGTTCGGAGACG	
ZQL83	GAGTGAGAGCCGCTGTGC	Amplification of *dptE* ORF for RT-qPCR
ZQL84	GGTGTCCCGTACGAGAACC	
ZQL85	CCAGATCCTCTCGACGGTG	Amplification of *dptM* ORF for RT-qPCR
ZQL86	CCGATACAGGCGCGTACC	
ZQL87	TTCCGGTACGAGCGGCTG	Amplification of *dptD* ORF for RT-qPCR
ZQL88	CGTCAGATCGAAGCGGCG	
ZQL89	TCCGCACCATCACGTTCAC	Amplification of *dptH* ORF for RT-qPCR
ZQL90	GACTTCCTGGGCCACCTG	

ZQL93	GGCCAGGATCGTGACGTC	Amplification of *dptR2* ORF
ZQL94	CGGAACGGCAGGAGTTCATC	for RT-qPCR
ZQL115	GACAGATCGCCGAGTTCGT	Amplification of *dptF* ORF for
ZQL116	GACGCTCCACAGCAGCTC	RT-qPCR
ZQL117	CTACCACGTGACCGTCAAGG	Amplification of *dptI* ORF for
ZQL118	GTCGTCGAACTCGTTTCCCTC	RT-qPCR
ZQL119	CAACGACGGCAGCTACCTC	Amplification of *dptG* ORF for
ZQL120	GGCGCATATCGGTCCAGTTC	RT-qPCR
ZQL121	CAATCCGCCGTACCAGGC	Amplification of *dptP* ORF for
ZQL122	GGACACGCCCGCTGTTGG	RT-qPCR
GJ91	CCAAGGGCTACAAGTTCTCC	Amplification of *hrdB* ORF
GJ92	TTGATGACCTCGACCATGTG	for RT-qPCR and probe *hrdB* for EMSA
CQ49	ATGGCCGGACAACCCACC	Amplification of probe *adpAp*
CQ50	CTGGCTCATGACGCTAAGC	for EMSA
ZQL39	GGCGTGGAACATACTGGCG	Amplification of probe *dptEp*
ZQL40	GCACAGCGGCTCTCACTC	for EMSA
ZQL43	CGAGGTCACCGCGTGGAT	Amplification of probe *dptAp*
ZQL44	GTCCATCTCTCGCGGATCC	for EMSA
ZQL51	CCGTTCCGATGCGAGTGC	Amplification of probe *dptR2p*
ZQL52	CGTGCAGGAAGGTGTTCGC	for EMSA
ZQL37	TCCCGGGAATCGCGAGAG	Amplification of probe *dptR3p*
ZQL38	GAAGAGGCGGAGGATGCG	for EMSA
ZQL47	GCGCGGTCAACAAGATTCTT	Amplification of probe *dptIp*
ZQL48	CCTTGACGGTCACGTGGTAG	for EMSA
ZQL57	CGGTCCGAACCGGCTCTTG	Amplification of probe *dptP-M*
ZQL58	AGGCGCTGCGGATCGATG	for EMSA
CQ61	ACCCGCCTTCCCTGACCC, 5' FAM	Amplification of *adpAp* for
CQ62	CCTGGCTCATGACGCTAAGC	DNase I footprinting
CQ63	CGCACCGGACGCCCTGAC, 5' FAM	Amplification of *dptEp* for
CQ64	GGCACAGCGGCTCTCACTC	DNase I footprinting
CQ65	CCGTTCCGATGCGAGTGC	Amplification of *dptR2p* for
CQ66	AACAGCCGGTGAGTGTGC, 5' FAM	DNase I footprinting
CQ67	CGAGGTCACCGCGTGGAT, 5' FAM	Amplification of *dptAp* for
CQ68	GGTTCGAGGATCTGCAGCAG	DNase I footprinting
Oligo dT-anchor primer	GACCACGCGTATCGATGTCGACTTTTTTTTTTTTTTTTV	For 5' RACE
Anchor primer	GACCACGCGTATCGATGTCGAC	For 5' RACE
CQ72	GGGGACGCTCCACAGCAG	dptA-SP1 for 5' RACE
CQ73	CGCAGAGGGCAACTCCGG	dptA-SP2 for 5' RACE
CQ74	GATCCGGTCGGGTGTCTC	dptA-SP3 for 5' RACE
ZQL69	GACGTTCAGCGAGCCCAG	dptR2-SP1 for 5' RACE
ZQL70	GCGTCGTAGGCGGAAGCG	dptR2-SP2 for 5' RACE
ZQL71	CGCGATAGGGGAGGGTGACG	dptR2-SP3 for 5' RACE

* Underlining: sequence of restriction enzyme shown in parentheses.

References

1. Macneil, D.J.; Klapko, L.M. Transformation of *Streptomyces avermitilis* by plasmid DNA. *J. Ind. Microbiol.* **1987**, *2*, 209-218.
2. Bierman, M.; Logan, R.; O'Brien, K.; Seno, E.T.; Rao, R.N.; Schoner, B.E. Plasmid cloning vectors for the conjugal transfer of DNA from *Escherichia coli* to *Streptomyces* spp. *Gene* **1992**, *116*, 43-49.
3. Li, L.; Guo, J.; Wen, Y.; Chen, Z.; Song, Y.; Li, J. Overexpression of ribosome recycling factor causes increased production of avermectin in *Streptomyces avermitilis* strains. *J. Ind. Microbiol. Biotechnol.* **2010**, *37*, 673-679.
